# A Case of Hemolytic Anemia With Acute Myocarditis and Cardiogenic Shock: A Rare Presentation of COVID-19

**DOI:** 10.7759/cureus.10657

**Published:** 2020-09-25

**Authors:** Ravi Singhavi, Kamal Sharma, Hardik D Desai, Rahul Patel, Dhigishaba Jadeja

**Affiliations:** 1 Cardiology, Krishna Shalby Hospital, Ahmedabad, IND; 2 Cardiology, U N Mehta Institute of Cardiology & Research Centre, Ahmedabad, IND; 3 Graduate Medical Education, Gujarat Adani Institute of Medical Sciences, Bhuj, IND; 4 Graduate Medical Education, Gujarat Medical Education & Research Society Medical College, Gandhinagar, IND

**Keywords:** acute myocarditis, anemia, cardiogenic shock, covid-19

## Abstract

Coronavirus disease 2019 (COVID-19) cases are on the rise globally, and mortality- and survival-related data are emerging every day. In addition, upcoming reports are suggestive of increased risk of cardiac ailments in high-risk patients. In the context of cardiac involvement, acute myocarditis has become one of the unexplored areas in COVID-19 patients, which could influence the long-term outcomes. In this report, we present a rare case that warrants further study on the subject due to the paucity of data in the literature. To date, no case of severe hemolytic anemias with stress cardiomyopathy/acute myocarditis in a patient of COVID-19 has been formally reported in the literature. The bedside echocardiogram had shown a possibility of acute myocarditis. The patient’s marked left ventricular (LV) functional recovery without coronary intervention further corroborates the same. Clinicians should be aware of the diversity of cardiovascular/hematological complications, as well as focused cardiac ultrasound study and the importance of echocardiography as a good screening modality for cardiovascular and hematological complications of COVID-19 infection.

## Introduction

As of July 23, 2020, the novel causative virus - the severe acute respiratory syndrome coronavirus 2 (SARS-CoV-2) - has affected 14,971,036 people and caused 618,017 deaths, with a case fatality rate (CFR) of 4.12 globally [1]. Recent studies have shown that mortality in the Takotsubo syndrome (TTS) variant of myocardial involvement in coronavirus disease 2019 (COVID-19) has been higher than mortality in TTS without COVID-19 and it affects both genders almost equally [2]. In this report, we present a case of acute hemolytic anemia with acute myocarditis and cardiogenic shock in a male patient with COVID-19 infection.

## Case presentation

A 20-year-old male presented to the emergency department with a one-day history of low-grade fever that had peaked to 101 °F just the night prior to the hospitalization with minimal flu-like symptoms. Physical examination on presentation showed blood pressure of 90/60 mmHg, heart rate of 120/minute, respiratory rate of 30 breaths/minute, and 92% oxygen saturation on room air. The patient had no significant past or family medical history. Laboratory tests on day one showed an elevated C-reactive protein (CRP) of 26 mg/L, erythrocyte sedimentation rate (ESR) of 75 mm for one hour, and elevated serum glutamic-pyruvic transaminase (SGPT) of 213 IU. On day two of admission in the COVID-19 intensive care unit (ICU), laboratory findings returned with a hemoglobin of 1.9 gm%, total white cell count of 22,000/cmm with a reduced platelet count of 44,000/cu mm. Peripheral smear showed normocytic hypochromic anemia with few fragmented RBCs and schistocytes with reticulocytosis with the possibility of hemolytic anemia due to smear features of fragmented RBCs. Lactate dehydrogenase (LDH), which is often a surrogate marker of both hemolytic anemia and inflammation, was elevated at 960 mg%. Elevated cardiac biomarkers, viz. troponin I of 9,565.2 ng/L and brain natriuretic peptide of 8,000 pg/ml, were also recorded. Lactate was 7.15 mmol/L and serum creatinine was 0.89 mg/dl.

**Figure 1:**
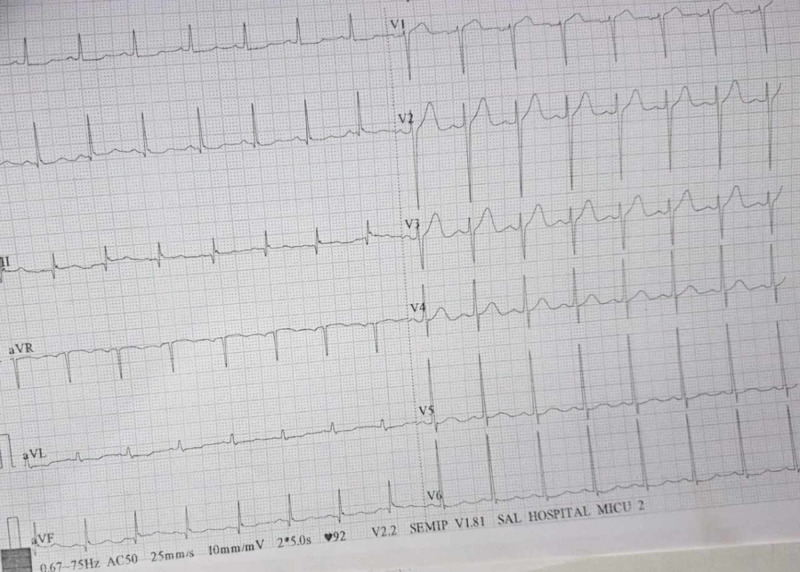
Electrocardiography showing ST-T wave changes in all leads with small Q in lead lll and aVF aVF: augmented vector foot

**Figure 2:**
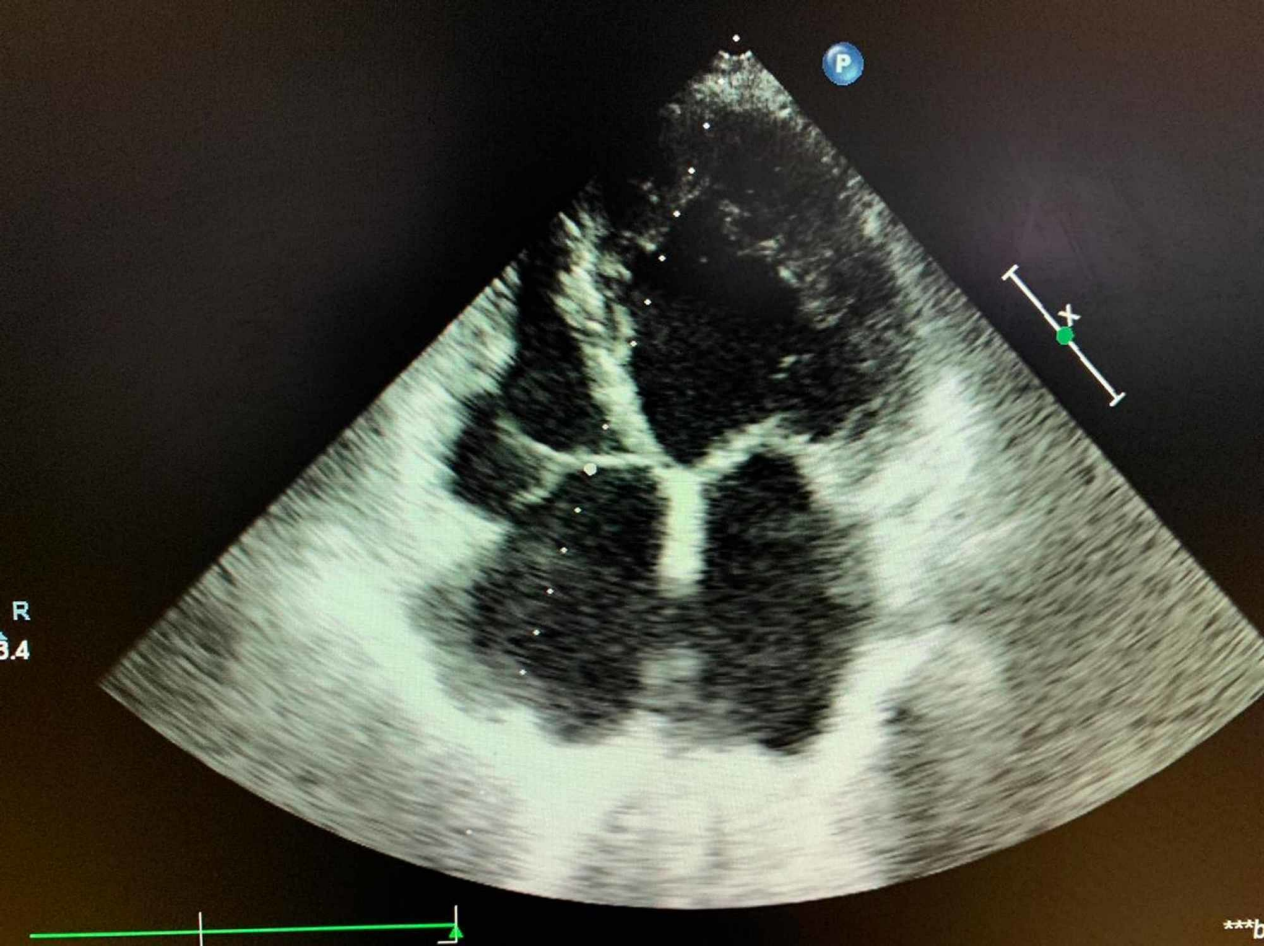
Echocardiography shows global hypokinesia, with preserved wall thickness

Electrocardiogram was mimicking of acute coronary syndrome, showing mild ST depression and T wave inversion (Figure 1). Echocardiography revealed global hypokinesia, with a preserved wall thickness (Figure 2), and left ventricular ejection fraction (LVEF) of 30%. High-resolution CT thorax (Video 1) and CT of abdomen-pelvis were unremarkable. The patient was managed in the ICU on inotropes. COVID-19 was strongly suspected despite normal high-resolution CT. Differential diagnoses included acute coronary syndrome, sepsis, acute fulminant myocarditis, and vasospasm in the setting of COVID-19 infection.

A nasopharyngeal swab was positive for high viral load (by cycle time) for SARS-CoV-2 by reverse transcription-polymerase chain reaction (RT-PCR). Given his positive COVID-19 test and hemoglobin of a mere 1.9 gm%, the decision was made to defer coronary angiography. The patient was transfused pack cell volume to correct anemia along with IV noradrenaline infusion, low molecular weight heparin, IV vitamin K, and a low dose of diuretics and steroids in the form of injection methylprednisolone pulse therapy. Clinically, the patient developed cardiogenic shock during the course of hospitalization and required up-titration of norepinephrine. He stabilized over the next two to three days and was finally discharged on day 10 with a hemoglobin level of 8.5 gm%.

**Video 1:** CT thorax showing cardiomegaly with congestive changes in lung parenchyma and no focal abnormality in lung CT: computed tomography

## Discussion

COVID-19 has been primarily a respiratory disease, but many studies have reported that it affects multiple systems. Cardiogenic shock due to myocarditis has been extensively reported as among the most common cardiovascular complications of COVID-19 [3]. Previous studies have reported that angiotensin-converting enzyme 2 (ACE2) receptors mediated effects on lungs, kidneys, heart, vascular endothelium by downregulating their expression and enhanced vasoconstriction with deleterious effects of the unopposed reticuloendothelial system [4]. It is also likely that virus-mediated immune response can cause consumption coagulopathies, acute hemolytic anemias, and other blood cell dyscrasias [3,5-6]. Cardiac findings of previously published autopsy series of patients with COVID-19 have reported cell necrosis without lymphocytic-myocarditis with no evidence of direct viral cytotoxicity [7]. Both acute myocarditis and acute hemolytic anemias and consequent severe anemia can together or alone produce acute heart failure and dilated LV/poor LV function.

In this report, we presented a case of a COVID-19 patient who developed acute myocarditis and severe acute hemolytic anemia, as evident from peripheral blood smear showing schistocytes (fragmented RBCs) in peripheral smear with acute severe anemia along with elevated LDH, which is also a surrogate marker for hemolysis. Acute heart failure with cardiogenic shock with possible stress cardiomyopathy is often characterized by transient severe global LV dysfunction in the absence of significant coronary artery disease. The cardiogenic shock was diagnosed based on the Intraaortic Balloon Pump in Cardiogenic Shock II (IABP-SHOCK II) definition: systolic blood pressure of 90 mmHg that requires more than 30 minutes of inotropic support. Exclusion of sepsis was supported by the normal value of serum procalcitonin apart from the corroborative echo finding of severe LV dysfunction [8]. In this patient, despite low oxygen saturation, the CT scan was clear, and the low saturation could be explained by central oxygenation impairment apart from peripheral vasoconstriction due to low cardiac output and inotropes that were being administered to the patient. PaO_2_ and SaO_2_ correlation varies more widely below 95% saturation and hence may be misleading. Severe acute myocarditis may sometimes manifest with low forward output and minimal pulmonary congestive manifestations [9]. It is hypothesized that high catecholamines, exaggerated inflammatory/immune-mediated response, and direct viral cytotoxicity and consequent effects of acute anemia (high CO state) may be the mechanism behind the development of such reversible transient stress cardiomyopathy secondary to acute heart failure due to acute myocardial damage and/or rapid RBC breakdown [10].

To date, no case of severe hemolytic anemias with stress cardiomyopathy/acute myocarditis in a patient of COVID-19 have been formally reported in the literature. In our patient, the bedside echocardiogram had shown a possibility of acute myocarditis. The patient’s marked LV functional recovery without coronary intervention further corroborates the same.

## Conclusions

It is anticipated that as the number of COVID-19 cases rises worldwide, there will be an increase in the number of associated cardiovascular manifestations and myriad complications. Clinicians should be aware of the diversity of cardiovascular/hematological complications and focused cardiac ultrasound study and critical care echocardiography as good screening modalities for cardiovascular complications of COVID-19 infection.
